# Supplementary material for: Influence of Advanced Organ Support (ADVOS) on Cytokine Levels in Patients with Acute-on-Chronic Liver Failure (ACLF)
Source: J Clin Med. 2022 May 15;11(10):2782. doi: 10.3390/jcm11102782 (PMC9144177; doi:10.3390/jcm11102782)
Supplement: Supplementary file 1 [file jcm-11-02782-s001.zip › jcm-1718183-supplementary.pdf]

Supplementary Table S1. Detoxification efficiency of the ADVOS treatment of bilirubin and regular laboratory parameters as assessed by standard laboratory in the clinical routine.

		Bilirubin (mmol/L)	Creatinin (mmol/L)	BUN (mmol/L)	Potassium (mmol/L)	Sodium (mmol/L)
**ADVOS**	Before	23.4 (17; 34.7)	2.75 (2.3; 4.6)	43 (35; 71.3)	4 (2.9; 5.2)	136 (130; 143)
	After	22 (17.8; 33)	3.3 (2.1; 3.8)	32 (22.8; 60.5)	3.9 (3.4; 4.3)	133 (130; 138)
	p-value	0.65	0.98	0.18	0.78	0.49

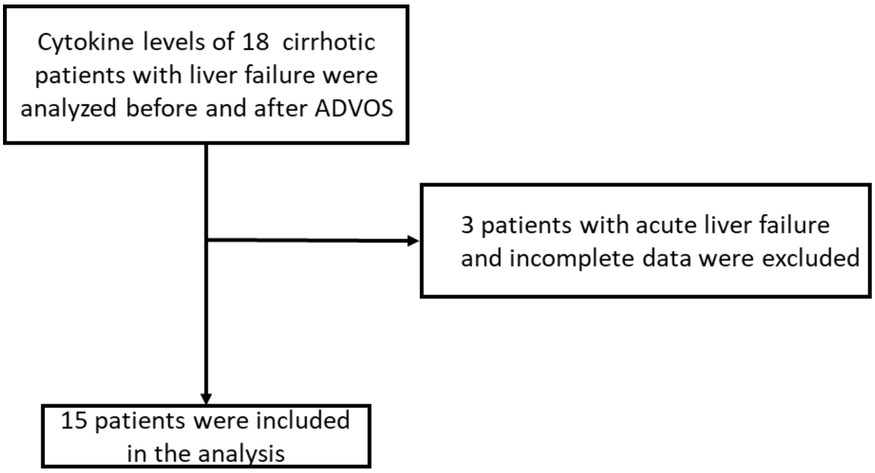

Supplementary Figure S1: Flowchart of the study.

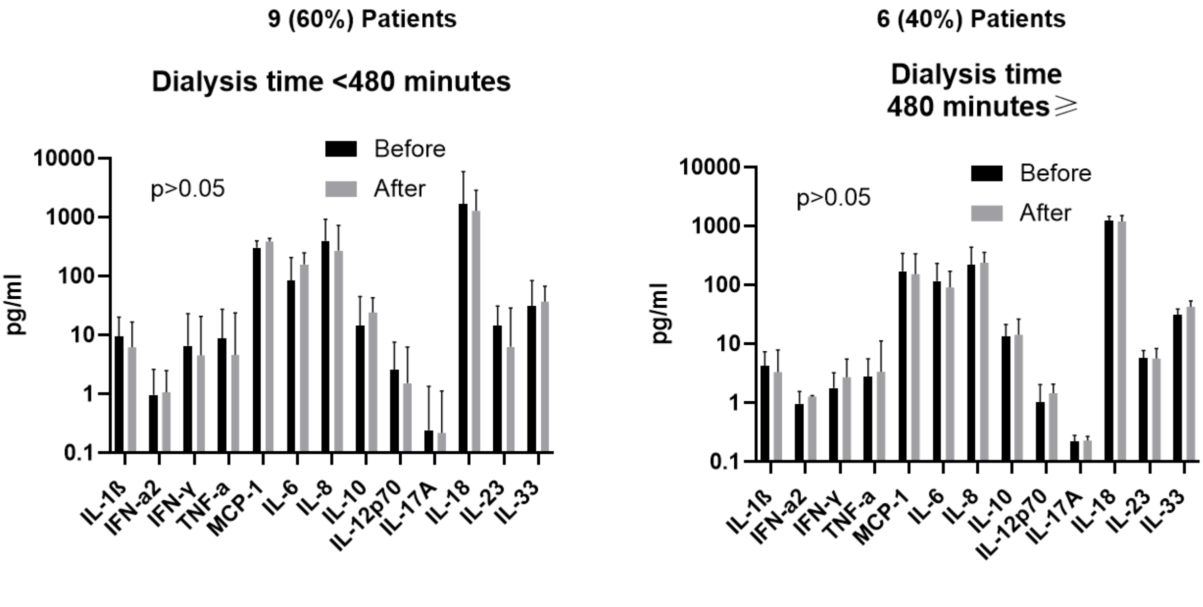

Supplementary Figure S2: Concentration of cytokines before versus after ADVOS treatment in patients with ACLF stratified for dialysis time as quantified by multiplex analysis.

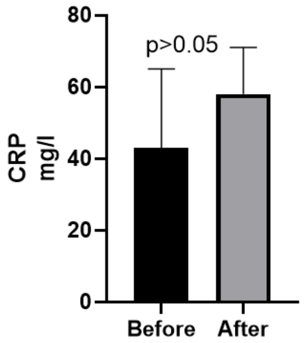

Supplementary Figure S3: Level of C-reactive protein (CRP) before versus after ADVOS treatment as assessed by standard laboratory.

Supplementary Table S2. Seize and isoelectric point comparison of selected cytokines versus albumin as reference (PhosphoSitePlus, can be found under https://www.phosphosite.org/homeAction, accessed on 4 February 2022).

	Isoelectric point	Dalton	Size versus albumin (%)
MCP-1	9.40	11,025	16
IL-8	9.10	11,098	16
IL-17A	8.82	17,504	25
IL-10	8.19	20,517	30
IL-23	6.02	20,730	30
IFN-a2	6.32	21,550	31
IL-18	4.54	22,326	32
IL-6	6.17	23,718	34
IL-12p70	6.21	24,874	36
TNF-α	6.44	26,000	37
IL-1beta	4.70	30,748	44
IL-33	8.9	30,759	44
IFN-γ	9.50	45,000	65
Albumin	5.92	69,367	100
